# “This is Why We All Show Up”: How Supporting Youth Cultivates Hope, Purpose, and Well‐Being of Adult Mentors

**DOI:** 10.1002/jcop.23182

**Published:** 2025-02-02

**Authors:** Helen Lee, Janelle Salcedo, Katharine Chen, Amy J. Anderson

**Affiliations:** ^1^ foundry10, Seattle Washington USA; ^2^ College of Education University of North Texas Denton Texas USA

**Keywords:** adult women mentors, mentor development, mentoring relationships, purpose, relational mentoring, well‐being, youth program mentors

## Abstract

This study explores the development of adult mentors participating in a summer youth program, focusing on how mentoring relationships and shared activities with youth impact mentors' personal and professional growth. The analysis integrates focus group data and participant observations collected over the course of the weeklong program to identify key themes. Findings reveal that affirming mentor‐youth connections within the program's youth‐initiated, collaborative mentoring model enhanced mentors' sense of hope, purpose, and well‐being. However, role ambiguity, particularly around how to collaborate with other adults to support youth, generated challenges for mentors. These insights contribute to the understanding of adult mentor development and adjustment and provide practical recommendations for designing youth programs that foster growth for both mentors and mentees.

## 1 Introduction

Youth mentoring programs engage adult mentors to support young people in order to promote healthy developmental outcomes. While extensive research highlights the benefits of mentoring for youth (DuBois et al. 2011; Raposa et al. 2019), there is a growing need to explore how youth mentoring shapes the development and well‐being of adult mentors. Recent calls to address the crisis of social connection in communities (Office of the US Surgeon General 2023) highlight an opportunity to leverage youth mentoring to enhance social connectedness and community well‐being (Keller et al. 2020). Moreover, greater attention to the social contexts of youth mentoring is critical to addressing the impact of systemic inequities on marginalized individuals within these programs (Hagler et al. 2023; Weiston‐Serdan 2017).

Examining the well‐being of adult mentors aligns with these recent calls and with community psychology principles that emphasize promoting wellness and social justice within broader social and organizational contexts (Prilleltensky 2001). That is, a mentor's growth occurs in relation to others, organizations, and societal norms. Although youth mentoring organizations are primarily designed to support youth development, community psychology theory suggests they also hold the potential to foster the well‐being of adult participants through their relational and communal focus.

The purpose of the current study is to qualitatively examine how adult women mentors' well‐being and sense of purpose are influenced by mentoring in a youth program designed to center the experiences of racially, ethnically, and gender minoritized participants. The transformative, community‐oriented youth program utilizes formal group mentoring as well as informal mentoring among participating adults and youth.

### 1.1 Relational Cultural Theory and Relational Mentoring

Relational cultural theory (RCT) provides an informative framework for understanding the relational mentoring approach explored in this study (Fletcher et al. 2007). RCT is a feminist theory that emphasizes the role of vulnerability and cultural context in human development (Miller and Stiver 1997). In contrast to other human development theories that emphasize independence, RCT recognizes relationships with others as central to women's psychological well‐being. Building on these principles, the model of relational mentoring applies RCT's core tenets to mentoring, shifting from hierarchical, top‐down approaches to mutual learning and development (Ragins 2024). Although primarily applied to adult career mentoring, this model offers valuable insights into how adult mentors change from their relationships with youth.

The RCT model of relational mentoring posits that relational conditions (e.g., vulnerability, mutuality) and relational skills (e.g., authenticity) predict the behaviors and processes within mentoring relationships. These processes include mutual learning, shared expertize, and fluid power dynamics between mentors and mentees. In turn, these relational processes drive both immediate mentoring outcomes (e.g., new knowledge, zest, empowerment) and broader relationship outcomes across multiple levels of analysis, including mentor, mentee, and organizational outcomes. The interplay of relational conditions, skills, processes, and outcomes are moderated by contextual factors, such as organizational norms and societal‐level gender and power dynamics.

Three tenets guide the RCT Model of Relational Mentoring (Fletcher et al. 2007). First, *interdependent self‐in‐relation* emphasizes that mentoring relationships are bidirectional, where both mentors and mentees influence one another's growth. In youth mentoring, this means that adult mentors can learn and grow alongside their mentees. Second, *growth‐fostering interactions* in relational mentoring have fluid boundaries, requiring vulnerability, empathy, authenticity, and emotional competence. These interactions lead to outcomes that extend beyond typical individual success to include zest for life, empowerment, self‐awareness, self‐worth, and enhanced relationships (Miller and Stiver 1997). Lastly, *systemic power* shapes how mentors and mentees relate to one another through shared experiences and what individuals take away from mentoring. Although RCT originally critiqued gender socialization, the RCT Model of Relational Mentoring addresses other power dynamics, such as class and race/ethnicity, that impact mentoring relationships.

These concepts offer valuable insights into how mentors are influenced by mentoring youth. Despite power imbalances between adults and youth due to age and other social identities, studying mentor benefits illustrates the bidirectional and power‐sharing nature of mentoring. Namely, mentors can be impacted by youth just as adults impact young people. Additionally, systemic power (e.g., race, gender) as a moderating factor aligns with youth mentoring scholarship emphasizing the role of social context and mentor‐mentee identities (Albright, Hurd, and Hussain 2017; Sánchez et al. 2013; Weiston‐Serdan 2017). The systems of privilege and oppression that youth navigate impact their needs and experiences within the mentoring relationship (Weiston‐Serdan 2017). Research has suggested that shared racial/ethnic identities between mentors and mentees can facilitate mentoring relationships development (Garraway and Pistrang 2010). However, these findings are not conclusive and may depend upon differing outcomes of interest. For example, shared race between mentor‐mentee demonstrated earlier initiation of alcohol use, yet girls of color with racially similar mentors reported better self‐worth over time compared to girls with white mentors (Rhodes et al. 2002). While all mentoring relationships have the potential to promote positive outcomes, shared identities may offer unique opportunities for validation, modeling, and the exchange of shared experiences (Albright, Hurd, and Hussain 2017). Indeed, the RCT Model of Relational Mentoring provides a useful framework for understanding how women mentors are impacted by their mentoring relationships in a youth organization primarily serving girls, nonbinary, and gender expansive youth of color.

### 1.2 Mentor Outcomes and Organizational Support in Youth Mentoring Programs

Research has examined how mentors develop through their involvement in youth mentoring programs, and the way organizational support may influence mentor experiences. A systematic scoping review of 96 studies on mentor development in youth programs found that most evidence focuses on the professional development and relational skills growth of mentors (Anderson and DuBois 2023). Fewer studies reported changes in mentors' cultural humility, mental health and well‐being, personal growth, and sense of contribution to others. Additionally, although present, few studies reported growth in identity, cognition, and physical health domains. Notably, most research concentrated on undergraduate mentors (18–22 years old) who work with youth for college course credit, with less evidence on mentor development in community‐based organizations. These findings underscore the need for further research on mentors in middle adulthood and mentors outside of higher education.

Prior studies support the current investigation into mentors' well‐being and sense of purpose within a youth program designed to foster a transformative space of belonging for mentors and youth. For example, mentoring has been linked to increased feelings of efficacy and empowerment to create positive community change (Andersen and Wellen 2023). Adult mentors in formal mentoring programs report enhanced sense of community and belonging, driven by their interactions with youth and other adults in the program (Graham and Jefferson 2019; Haddock et al. 2013). For instance, program activities may facilitate an exchange of lived experiences and provide spaces for mentors to learn from and connect with one another. Additionally, mentoring can also offer an opportunity for relief from stressors in mentors' everyday lives and foster a greater sense of purpose and fulfillment (Graham and Jefferson 2019; Haddock et al. 2013; Morse et al. 2022). These experiences allow mentors to feel positively about their lives and connect to others around them.

Research indicates that program conditions play a key role in influencing mentors' well‐being and purpose. The guiding framework for this paper, the RCT Model of Relational Mentoring, suggests that growth‐fostering interactions, such as shared vulnerability and mutual learning, are facilitated by organizational norms and climate (Fletcher et al. 2007). Research has identified the significance of program features and quality in shaping mentors' experiences. For example, some mentors benefit from structured activities that clarify their roles, while other mentors prefer more flexibility (Weiler et al. 2014). Additionally, the quality of mentor‐mentee relationships also matters. Stronger connections are associated with greater personal growth among mentors (Graham and Jefferson 2019; Morse et al. 2022). In this way, program structure and relationship quality can shape how adults experience youth mentoring.

Organizational support is also influential. Meta‐analyzes of formal mentoring programs show that ongoing support and training have large effects on program outcomes (DuBois et al. 2002). Training helps mentors develop the skills to support youth mentees, while supportive staff relationships enhance mentors' experiences (Anderson et al. 2023; Keller et al. 2023; Kuperminc and Deutsch 2021). Both forms of support likely influence mentors' experiences by bolstering their self‐efficacy, which is linked to their perceptions of relationship quality (Deane et al. 2022; Strapp et al. 2014). Consistent with the RCT model of relational mentoring, youth mentoring research suggests that the contextual environment (i.e., structure and quality of program activities, organizational support) shapes how mentors' well‐being and sense of purpose evolves through youth mentoring experiences.

### 1.3 Current Study

Informed by the RCT model of relational mentoring, the current study sought to examine the development of adult mentors in a summer youth program. Although prior theory and research suggest that adult mentors change through the experience of mentoring youth, existing research has primarily focused on young adults who mentor through their university (Anderson and DuBois 2023). Research on mentors' in middle adulthood who participate in a different youth mentoring context can contribute to the literature by expanding understanding of how mentoring impacts the well‐being of mentors, and what program conditions might influence this development. To address these aims, the current qualitative study explored the following research questions within a summer youth program:

1. How did mentoring impact mentors' creative development (willingness to express one's self and ideas; willingness to try new/unfamiliar mediums and take creative risks)?
2. How did mentoring impact mentors' social‐emotional development (self‐awareness, communication, and other interpersonal skills) and wellbeing (emotional state; sense of psychological safety; feelings of anxiety and self‐esteem)?
3. How did mentoring impact mentors' identity development (sense of self) and sense of community (sense of belonging, feelings of affirmation)?

## 2 Methods

This research project examined the development and experiences of adults mentoring in a summer youth program. Multiple data sources were collected, triangulated, and iteratively analyzed to better understand the impact of the mentoring experiences on participants.

### 2.1 Program Description

Young women empowered (Y‐WE) is a nonprofit youth organization that “cultivates the power of diverse young women^1^ to be creative leaders and courageous changemakers through transformative programs within a collaborative community” (Young Women Empowered n.d.). In the 2022–2023 school year, Y‐WE served over 1100 community members and offered 22 programs and events. The organization offers experiential learning opportunities through school‐based and out‐of‐school summer programming as well as one‐time events. All programs and events are free to attend and open to young women, trans, non‐binary, and gender expansive youth (ages 13–26). These programs typically incorporate youth voices through youth‐initiated activities and scaffolded leadership opportunities.

Each Y‐WE program provides adult mentors with opportunities to support the personal and professional development of youth participants. Y‐WE welcomes women from all backgrounds as mentors, intentionally recruiting and prioritizing those whose backgrounds and experiences reflect those of youth participants. All interested mentors must complete an application, background check, and interview in order to serve as a mentor in the program. Interviews typically assess alignment of mentors' background and interests with the goals of the organization and programs of interest. This alignment contributes to a nurturing environment that is attuned to the specific needs and experiences of youth participating in Y‐WE programs. To make volunteering more accessible, mentors are offered an equity stipend of approximately $100 a day at the end of the program to help offset costs, such as time away from work or other responsibilities.

The current project focused on Y‐WE create, a week‐long summer camp designed to support youth in exploring various artistic mediums and engaging in creative risk‐taking. The daily camp schedule included an open plenary, lunch, track‐time, and a closing plenary. In the summer of 2023, create offered three tracks to which youth could apply: ceramics, sewing, and cultural kitchen. each track was led by one to two adult facilitators and supported by two to three adult mentors. Two other adult facilitators led the overall camp experience, primarily the opening and closing plenaries, and four Y‐WE staff members led program logistics and provided support as‐needed (e.g., sign‐in, lunch orders and distribution, supplies, family communication).^2^

Create camp involved multiple mentoring approaches (Kuperminc and Deutsch 2021). First, mentors supported youth in their assigned track akin to group mentoring. Outside of track time, mentor‐mentee matches occurred during planned plenary activities and informally before the start of the program each day and during lunch. These interactions might involve one mentor and one youth, one mentor and many youth, or multiple mentors and one youth. Mentoring was often initiated by youth across these situations and involved the guidance and support of multiple mentors.

Before camp began, Y‐WE create mentors participated in a training with Y‐WE staff that covered risk management policies and introduced them to the collaborative mentoring model integral to all Y‐WE programs. To help mentors understand their roles within this approach, the training covered best practices for setting healthy boundaries, responding to sensitive topics, building authentic connections with youth, and creating accessible spaces. During camp, mentors participated alongside youth in daily activities, helping them stay attuned to individual needs. Program staff debriefed with mentors at least once during the camp to understand and address any program challenges, and some track facilitators elected to also meet with mentors on this same topic.

### 2.2 Procedures

Eight adult mentors (including the first and third authors) volunteered throughout the entire week of the 2023 create program (two in ceramics, three in sewing, and three in cultural kitchen). Two of the four Y‐WE staff also provided mentorship within program tracks throughout the entire week (although to a lesser extent than the eight adult mentors because of their program coordination responsibilities), and so were included in the focus group invitations. All adult mentors were invited to participate in focus groups during create camp and a member checking process after the research team systematically analyzed the data. All eight mentors (who were not researchers), including the two Y‐WE staff members who assisted with mentorship, agreed to participate in the focus groups, and five of these same eight mentors participated in the member checking process.

Semi‐structured focus groups aimed to explore mentors' experiences, development, and attitudes throughout the program. Observations sought to help researchers understand the program structure and environment. Observations also aimed to provide opportunities for researchers to observe mentor and youth interactions and behaviors that could inform focus groups, as well as confirm or expand themes that emerged from focus groups. Member checking was conducted to incorporate participant feedback on emerging themes.

### 2.3 Focus Groups

The same mentors were interviewed twice–during lunch on the third day and final day of the week‐long program. Each focus group session was approximately 45 min long, led by either the first or third author, and facilitated in a private space with no more than four participants in each group to encourage open sharing and discussion. One mentor was unable to attend the entire time of the final scheduled session and so was later interviewed one‐on‐one by the third author after camp concluded that same day. Therefore, a total of five sessions were completed.

The focus groups explored mentors' experiences as well as the impact of mentoring in the program on their social, emotional, and creative development and overall well‐being. The focus groups also examined challenges faced, insights gained, connections with youth, and suggestions for improving mentor support. Questions included: “How has your perspective on mentoring changed this week if at all?,” “Tell me about a moment where you connected with a youth,” and “What is one change that would help you better fulfill your role as a mentor at camp?” (see Table 1 for the full list of focus group questions).

**Table 1:** Focus group questions.

Day	Main questions
3	1. Tell me about an impactful experience you've had at create so far. a. How did this experience impact you? 2. What challenges or uncertainties have you encountered if any at all? 3. How has your perspective on mentoring changed this week if at all? 4. What is one change that would help you better fulfill your role as a mentor at camp?
7	1. What have you learned from your experiences as a mentor this week? 2. Tell me about a moment where you connected with a youth. 3. What additional challenges or uncertainties have you encountered this week, if any at all? 4. What is one change that would help you better fulfill your role as a mentor at camp?

Focus group sessions were audio‐recorded and transcribed by a professional service, with research team members reviewing transcripts for accuracy and to remove identifying information. Participants received $50 in the form of an electronic gift card or payment for completing the focus group sessions.

### 2.4 Observations

The first and third authors conducted daily observations while also participating in the program as mentors. Following a structured guide, they observed interactions and engagement relevant to the research questions (see Table 2). The guide also prompted observations of the program structure and facilitation. Researchers debriefed daily to refine focus and clarify any questions. At the end of each day, they recorded their observations based on the prompts in the guide.

**Table 2:** Excerpt of observation guide.

Category	Includes	Researchers should note
Social anxiety/fears	Feelings of nervousness/shyness/uncertainty around others Difficulties talking to others/participating in activities	What participants say/do Who engages, who doesn't What mentors say/do Factors that may encourage/discourage participation and engagement Any changes in behaviors and antecedents
Social‐emotional development	Developing self‐awareness (e.g., instances in which participants are reflecting, giving or receiving feedback, practicing gratitude, examining emotions, and/or practice mindfulness) Developing communication/interpersonal skills (e.g., participants are actively listening, collaborating with others, showing empathy, proactively communicating needs)	What participants say/do Who engages, who doesn't What mentors say/do Factors that may encourage/discourage participation and engagement Any changes in behaviors and antecedents
Wellbeing	Presence of positive emotions (e.g., joy, love, interest, awe). Absence of negative emotions (e.g., fear, loneliness, sadness). Satisfaction with work (e.g., pride in process/product, feeling engaged with activities). Positive functioning (e.g., engaging with others, trying new activities, overcoming challenges) Physical wellbeing (e.g., feeling healthy, feeling energized).	What participants say/do What activities/practices encouraged/discouraged wellbeing
Identity and community	Forming a clear and unique view of one's self Being happy and comfortable with one's self Feeling a sense of belonging Feeling affirmed in one's identities (e.g., youth see themselves in their mentors, youth's complex and intersectional identities are centered in the program)	What participants say/do What activities/practices encouraged/discouraged identity development How identity is affirmed/centered What mentors say/do
Creative development	Expression of one's ideas, feelings, thoughts Engaging in artistic activities Trying new/unfamiliar mediums and processes Expressing one's individuality through art Feeling supported to take creative risks	What participants say/do Who engages, who doesn't What mentors say/do Factors that may encourage/discourage participation and engagement Any changes in behaviors and antecedents

### 2.5 Member Checking

All eight mentors were invited to review and provide feedback on the authors' interpretation of the data. The five who agreed reviewed a draft of key findings and provided comments directly in the draft as well as in a Zoom meeting with either the first or second author. Following a protocol developed by the first author, the meetings focused on validating and expanding findings based on participants' experiences. Participants received an additional $100 for their participation in the member checking process. See Appendix A for more details.

### 2.6 Participants

All Y‐WE Create mentors were invited to participate in the study. All eight mentors, including two full‐time Y‐WE staff members who coordinated the program and served as mentors during the program, consented to observations and participated in focus group discussions.^3^ Five of these mentors participated in the member checking process. All mentors identified as women or gender minorities and people of color. Five had previously mentored in a Y‐WE program and one had participated in Y‐WE programs as a youth. Participants ranged in age from 26 to 37 years old (*M* = 32.1).^4^ See Table 3 for additional participant details.

**Table 3:** Description of participants.

Participant name	Returning/new mentor	Participated in focus group	Participated in member checking
Elizabeth	Returning	Yes	No
Anya	Returning	Yes	No
Alexis	Returning	Yes	Yes
Rhea	Returning	Yes	Yes
Shannon	Returning	Yes	Yes
Aishah	New	Yes	No
Stephanie	New	Yes	Yes
Jamila	New	Yes	Yes

### 2.7 Data Analysis

Following Braun and Clarke's (2006) thematic analysis framework, the data was initially examined and discussed by the first three authors to develop an understanding of mentors' experiences and development within the youth program. Codes were then generated to organize data into key themes and formed the initial codebook. The codebook was refined through iterative, collaborative discussions between all four authors to ensure alignment of analysis with research questions. Focus group data was then coded using the final codebook by all four authors.

Initial themes were generated from the coding process, which entailed grouping related codes and discussing overarching patterns and trends between codes. These themes were refined and validated through additional discussions amongst authors and cross‐checking themes with observation data. Themes were also further refined and validated through a member checking process, which encouraged interested participants to confirm or challenge the accuracy of themes relative to their experiences and perspectives.

The embedded role of the first and third authors as mentors and researchers, along with prior relationships between mentors, may have influenced group dynamics and findings. To address this, the research team held reflexive conversations throughout the process (e.g., discussed and documented potential biases and assumptions that may shape the analysis), coded for themes such as consensus, dissent, and expansion, triangulated focus group data with observation data, and validated interpretations through member checking.

### 2.8 Positionality

The first three authors met lead Y‐WE create staff before the 2023 program began. The first and third author partnered with Y‐WE to evaluate the 2022 create program, and the third author served as a mentor in the 2019 create program, where they connected with some of the staff, facilitators and mentors involved in the 2023 program. The first and second authors had no prior experience mentoring in Y‐WE programs before 2023, and the fourth author was only indirectly involved with Y‐WE through the analysis for this study.

The first three authors attended the mentor training and program onboarding sessions for the 2023 program. The second author attended in person, while the first and third authors completed an asynchronous option and connected with Y‐WE staff over email. The first and third authors also assisted with program setup the day before camp, meeting again with lead program staff and other mentors.

The varied interactions with Y‐WE and embedded roles of two research team members enabled the authors to collect detailed descriptive data and better contextualize interactions and reflections within a broader social and cultural context (DeWalt and DeWalt 2010; Lucas‐Thompson et al. 2021; Pryce et al. 2021). Positive prior interactions with participants and staff, coupled with a commitment to reciprocity, likely fostered trust and encouraged honest, detailed responses, enhancing the quality of the qualitative data (Cresswell 2013). Additionally, Y‐WE's organizational values and practices likely contributed to authentic engagement in the program and candid responses in the focus groups and member‐checking.

However, these connections and embedded roles may have introduced bias, potentially affecting the objectivity and generalizability of findings. Participants may have modified their behavior or responses due to their familiarity with researchers or due to their investment in Y‐WE (Maxwell 2013). While researchers' embedded roles contributed to rich data and interpretation quality, the team utilized multiple methods to ensure interpretations were not overly reliant on one perspective or source. Additionally, researchers engaged in reflection of biases and assumptions throughout the research process, ensured there were multiple opportunities for both participants and peers to challenge interpretations and help identify areas where bias may have influenced conclusions, and utilized a systematic and collaborative coding approach to minimize personal biases and the potential influence of a single researcher (Braun and Clarke 2006; Chase 2017; Miles, Huberman, and Saldaña 2019; Olsen 2004).

## 3 Results

Our analyzes of focus group sessions and program observation data revealed four key themes related to the growth and adjustment of adult mentors working within Y‐WE's youth‐initiated, collaborative mentoring model. Consistent with the RCT Model of Relational Mentoring—which emphasizes the mutual, bidirectional nature of mentoring relationships—we found that mentors experienced personal growth from their interactions with youth. First, meaningful and affirming connections with youth deepened mentors' sense of purpose and hope. Second, engaging in creative practices alongside youth in a supportive community encouraged mentors' agency and healing. However, mentoring did not come without difficulties: mentors expressed challenges in confidently supporting youth as they pursued their creative visions. Finally, Y‐WE's expansive and flexible mentoring model–while uniquely transformative for youth and mentors–created some role ambiguity, which led to uncertainty and stress for mentors at times. This resulted in the following four themes: (1) *Cultivating hope and purpose through affirming mentor‐youth connections*, (2) *Creative empowerment and healing through program participation alongside youth*, (3) *Challenges to creatively supporting youth participants*, and (4) *The expansive and ambiguous role of mentors*. These themes illustrate the complex, reciprocal nature of mentoring relationships and its impact on mentors' emotional and psychological growth, especially for mentors whose backgrounds and identities reflect those of youth.

### 3.1 “And This Is Why We All Show Up”: Cultivating Hope and Purpose Through Affirming Mentor‐Youth Connections

Mentors described how meaningful, affirming connections with youth strengthened their sense of purpose and encouraged feelings of hope. Many mentors shared that their involvement in Y‐WE Create allowed them to form deep connections with youth and other mentors, affirming their own identities and experiences and identities while also supporting the growth of youth participants. Witnessing and contributing to the development of youth participants emerged as a powerful source of hope and purpose for mentors.

For example, Alexis, a returning mentor, recounted two moments of connection with Camila, a youth participant new to Y‐WE. The first of these moments occurred during a group milling activity at the beginning of camp when Alexis was paired with Camila. The second time took place on the third day of camp, when Camila independently sought out Alexis to talk about their shared experiences. Reflecting on these moments, Alexis shared:

We actually have very similar upbringings… her dad is going to be the principal at her high school, and my dad was my principal in elementary school and middle school. And so talking about the pressures that we feel or felt being in that position, especially when both of our dads are folks of color, who are perceived in a very certain way, especially in Seattle public schools… it really surprised me… I don't often get those deep moments with youth where I can actually have a one‐on‐one conversation for 20 min… And it was in that moment where I was like, “This is it. And this is why we all show up.”

Alexis' first notable connection with Camila during the milling exercise–a whole group activity designed to initiate and develop connections among participants and prompt reflection on the program theme–encouraged Camila to reach out later, sparking a deeper conversation about their shared backgrounds and experiences. These moments of connection illustrate growth in both youth and mentor, showing the power of identity‐affirming connections. Alexis's declaration, “this is why we all show up,” underscores the importance of such connections for people of color and gender diverse individuals, who are often in environments where they feel unseen or unsafe to fully express themselves.

Another mentor, Shannon, expressed a similar sentiment during the member‐checking process:

I connected with a few of the youths who (are) Asian American like me. There was one young woman in particular who (is) Cambodian American like me and as our community is relatively small and (doesn't) have much representation in many spaces, we connected over the course of the week. I would have loved to see an older Cambodian American woman (as) part of my chosen activities and it means a lot that I could be that for someone else.

For Shannon, being a mentor who shares the same cultural background as the youth was a particularly powerful experience because she had never experienced this kind of representation in her own childhood and adolescence. This experience not only strengthened her sense of purpose but also allowed her to support youth in envisioning possibilities for themselves. Such representation can be empowering for youth and affirming for mentors, reinforcing their commitment to mentoring, as seen in the cases of Alexis and Shannon.

While mentoring youth with similar backgrounds hold special significance to mentors identifying as people of color, creating an inclusive space for all youth was equally impactful. Aishah, a new mentor to Y‐WE, described how this experience made her more hopeful and strengthened her commitment to supporting youth in her work as a school counselor:

Not being from here, but envisioning this space more as a concept in my head and not a physical space, but then being able to see it as a physical space that's functioning and so inclusive and just absolutely amazing made me emotional yesterday…I wish that every kid could have this, and it's so amazing that it is something here…I'm a school counselor, so I'm thinking about my kids…and I'm like, dude, if we just had something like this where my different kids would feel this love and community and all of those things…And for the ones who are a little bit more to themselves, there are still opportunities to share when it's just two or three. Or there are so many different modes of communication that happen that…everyone gets to leave with “I got to be seen.”

Aishah expressed amazement and feeling emotional from witnessing the “love and community” at camp. These “intentional” acts reminded her of the need and possibility for identity‐affirming environments–that being part of an inclusive and mindful environment where one is “seen” positively supports the social and emotional growth and well‐being of all people, and especially those who experience marginalization. Later in the same focus group, Aishah shared another meaningful moment connecting with youth:

(D)uring the gratitude circle at the end of the day…it's literally just (everyone) sharing…something that we're grateful for. And in a world where gratitude is so not appreciated or it's just not present a lot of the time, how specific some of the kids and mentors were being…it was just such an uplifting thing to hear and be a part of…

We're from so many different parts of the world and different pathways in life and whatever, but we can still come together and form a circle and share things that we're grateful for.

Being part of this identity‐affirming community reinvigorated Aishah's belief in the need for inclusive and supportive environments for all youth and inspired her to explore new ways to support her students as a school counselor. The thoughtfully designed and well‐facilitated activities provided her with fresh perspectives on fostering connections and inclusion.

Another significant contribution to mentors' sense of hope came from observing and supporting the creative, social and emotional growth of youth participants. These instances reinforced mentors' commitment to mentoring. Mentors emphasized that playing a positive role in the development of young people at Y‐WE inspired them and deepened their commitments to this work as well as to the Y‐WE community. In one of the focus groups that took place on the last day of the program, Anya, a returning mentor, talked about the growth of two youth in the cooking track:

There were two moments actually where I connected with a youth who is very soft‐spoken, and I am not a very soft‐spoken person, so I was not her go‐to mentor… But then today…her voice was louder, and she came up to me and was talking about what she was excited to do today. And it's like, “Are you the same person?” And it was so cool to be like, “Oh, sweet. My energy was not what you wanted. You needed something else to get comfortable.” And then when you got comfortable, you were like, “Okay, now I want your energy. Now I want to engage with how you engage in the world.” And that was just so freaking cool and such a testament to we do not need to force connection. We do not need to force, you know, “Come on, speak up, let's go.” Right? It is okay that it is a process. And I think in our society and in school a lot, they don't get the chance to just be in that space.

In the above reflection, Anya shared her observation of the social and emotional growth of a youth participant over the course of the weeklong program. Anya attributed this growth in part to Y‐WE's community mentoring approach, in which youth are not assigned mentors (or vice versa) but rather empowered to approach mentors as they wish. By facilitating a supportive environment for youth to determine which connections they want to make, Anya observed this youth doing so in the way that felt most appropriate for them.

To further clarify what made the Y‐WE program a supportive environment, Anya presented her experience with a different youth in the same track. In the exchange below, which continues from the previous excerpt, Anya attributed her consistency in being available to the youth as an important contributor to how the youth interacted within the program:

We had one youth who came just for 1 day. Her mom signed her up for camp, and so she was like hella pissy about that. She didn't make this choice herself. And it was kind of funny because every other mentor and facilitator in the track went and tried to connect with her, and she just shot them all down and… I was just like, “I'm not going to leave.” That was the only energy that I was putting out, was you could say whatever the hell you want to me, you could not talk to me at all. But the one thing that's going to happen here is I'm not going to go anywhere… And she just totally, not fully opened up immediately, but it was a very much like, “Oh, no matter how much I push you…You're still not going away.” And that was a really cool moment. (S)he was so upset about being here, and then she was jumping into making everything and cracking jokes…

We all wish that she ended up being here for longer than a day, but even just that day, I felt a really big shift in her. And I felt like she was able to take off a mask or two of protection…

According to Anya, this youth, who was new to Y‐WE and who only ended up attending for a day, initially showed disinterest in part because she did not choose to participate in the program—rather her mother had signed her up. Despite her initial apathy, this youth increasingly engaged in program activities throughout the course of the day. Anya attributed this attitude change to the practice of respecting boundaries among Y‐WE youth and mentors as well as mentors' consistency in being available to youth should they wish to engage.

Connections with youth that lead to observable growth, like the one Anya described, reinforced mentors' sense of purpose and the significance of their role within this community. For returning mentors, contributing to the growth of a young person over multiple programs seemed to deepen not only their commitment to this work but also their commitment to Y‐WE. Three of the returning mentors mentioned how playing a part in the growth of Kalani and Carmen, two Y‐WE Create facilitators who started as youth program participants, strengthened their commitment to supporting youth in Y‐WE programs. Anya, one of these returning mentors, said, “A really special thing is… watching Carmen run a program… I've watched them grow up really significantly in this program (and now) running one. And that's really awesome.” Within the same focus group, Alexis, another returning mentor, similarly expressed their pride and joy in witnessing Carmen's growth from youth program participant to program facilitator: “If I think too hard about it, I cry.”

These mentors highlight how “special” it is to witness the growth of a young person. While these mentors do not explicitly bring up their own involvement in supporting the youth in these excerpts, it is clear from other moments in the focus groups that they have been present for Carmen throughout their time in Y‐WE. Similarly, Shannon shared the impact of witnessing Kalani's growth from youth participant to program facilitator, which reflected the growth Shannon and Kat have seen in other youth participants of Y‐WE programs:

Shannon: (A)s the week progresses, you see the kids come out of their shell and volunteer for things and speak up and everything. That's always one of my favorite things about camp, and Y‐WE in general, just seeing how the young people grow throughout the programs and where they started, where they end up.

Like Kalani (camp facilitator) was a youth. (S)he was so quiet… I was in the Speak program with her, and she was a very quiet girl, but then she would spit this poetry, which was like…“Holy (expletive).”…(T)his is her first time (facilitating) a big program, but seeing her grow in the last 9 years or something, for me, has been a huge thing… (b)ecause she and I have always been close since the beginning. I feel like she's one of the youth that I've connected with.

Kat: Yeah. I feel that's a really cool part of Y‐WE is it's not just one program and the week, and you form those relationships, and they're…done. They really try and sustain that. So you can see the growth and so the youth can continue to connect and be supported.

Following Shannon's comments, Kat (a returning mentor and core study team member) similarly expressed awe at Kalani's growth as well as in recognizing the power of these mentoring relationships and moments of connection with youth. Both Kat and Shannon emphasized how the connections Y‐WE sustains can contribute to the development of youth participants in significant ways.

During the member checking process, Shannon further elaborated on her motivations for serving as a mentor over the years:

Seeing the youths' growth and development is one of my favorite “side effects” of being involved in Y‐WE. While I love being part of the different programs and the activities we do in the moment, seeing the bigger, long‐term picture of how this work impacts young people is the most rewarding. It makes me think of the saying, “It takes a village to raise a child.” I'm not a parent myself, but being part of Y‐WE has allowed me to be part of these young people's villages as a proud “auntie.” And I really value that community connection.

Mentoring at Y‐WE allowed mentors to forge genuine relationships and moments of connection with both youth and other adults. These moments of affirming connection supported the social, emotional, and creative growth of youth participants, whether in the short term during the week‐long Y‐WE Create program, or over the long term across multiple Y‐WE programs as with Kalani and Carmen. These connections also contributed to the growth and well‐being of mentors. Mentors expressed joy and appreciation from their experiences working with youth in the Y‐WE program, which became a source of hope for them and strengthened their sense of purpose in engaging in youth‐serving work.

### 3.2 “I Keep Coming Back Because This Is What My Younger Self Wanted”: Creative Empowerment and Healing Through Program Participation Alongside Youth

Since Y‐WE's mentoring model encourages mentors to participate in activities alongside youth participants, one aspect of the study investigated the impact of mentoring and program participation on mentors' creative growth and social emotional well‐being. We found that engaging in creative activities with youth increased mentors' sense of agency. Mentors also noted that these experiences felt healing because they did not have similar opportunities as children but were now able to support such opportunities for young people.

For Shannon and Elizabeth, participating in Y‐WE create allowed them to explore new artistic mediums–ceramics and sewing, respectively. Both mentors expressed excitement about learning new skills alongside youth participants. Elizabeth, a first‐time mentor at Y‐WE create, described her experience learning to sewing as empowering:

I just feel so excited about the sewing skill. It's a brand new skill for me and I was joking with my partner yesterday. I went home, I was looking at all my clothes, I was thinking of all these cute outfits I want to make and not finding clothes that fit great…I was like, “I can make so many clothes.” And then I was joking, “I feel less disenfranchised from the means of production.” So just that feeling of like, “I can do this thing for myself if I want to.” And I can see that in the youth too.

Learning to sew not only allowed Elizabeth to express herself creatively but also bolstered her confidence in a new skill. During the member checking process, Alexis and Jamila, both returning mentors and either current or former Y‐WE staff members, emphasized that a core goal of create is to foster agency and vision to create a better world not only among youth participants but also among mentors who engage in these creative practices.

For Shannon, the invitation to experiment with new artistic mediums was a major draw of mentoring at create. Shannon had mentored in multiple Y‐WE programs in past years, including as a facilitator, and noted that she often chose to mentor in create track areas unfamiliar to her. She described the experience of learning a new art form and creating at Y‐WE as personally healing:

I am here as a mentor, but it's also me, my younger self coming to camp (laughs). And that is also why, when I sign up for whatever track, it's always like, “Oh, I don't know anything about this.” So I personally can experience it myself… I've talked with a lot of other mentors (and we're) like yeah, I keep coming back because this is what my younger self wanted, would've loved, and it's healing for myself.

Shannon's reflection highlights the significance of accessing creative opportunities she missed as a child on her current well‐being. During the member‐checking process, she mentioned how, despite incredibly supportive parents, financial constraints limited access to such artistic pursuits during her childhood, making such opportunities now especially meaningful. Alexis, who also mentored in many past Y‐WE programs, echoed this sentiment, noting that many mentors in Y‐WE “express wishing they had a space like Y‐WE when they were younger.” Alexis further explained that the Y‐WE mentoring model aims to support healing amongst its community of youth and adult mentors: “The intergenerational space allows mentors to tap into their younger self while also holding space for young people.” Alexis added:

Reflecting back on my first experience with mentoring, it was powerful to have a mentor as a high school student. Would've been amazing to have a space like Y‐WE then. But (having been a part of Y‐WE as an adult) has been transformative and healing. Y‐WE provides a space for young people and community to heal. This is why I've stayed on to mentor after transitioning from staff.

Like Shannon and Alexis, Stephanie expressed that while she wished for programs like Create in her youth, being part of making them available to young people now is particularly healing: “I want the world to be better than how it was for me. That's why I'm getting into this.” This is reflected in what Jamila added during the member checking process: “Showing up and being your authentic self is encouraged (at Y‐WE) and is very healing for youth and adults.” These findings reinforce the importance of creative empowerment and community in not only cultivating hope and resiliency but also addressing the impacts of trauma that women or gender minorities and people of color are more likely to experience.

### 3.3 “I Just Don't Know How to Do It, So I Don't Know How to Help.”: Challenges to Creatively Supporting Youth Participants

Although mentors overwhelmingly had positive experiences participating and mentoring in Y‐WE summer programs, they still encountered challenges that left them questioning their roles or how to best support youth in the moment. Mentors in the ceramics and sewing tracks, for example, expressed uncertainty and occasional stress when they lacked the technical skills in which youth were seeking support or when they realized that youth needed more guidance, instruction, or time than could be provided within the week‐long program.

Elizabeth and Shannon both shared specific challenges around not having the technical skills to support youth's creative endeavors, highlighting a common experience for mentors in the ceramics and sewing tracks. Shannon, who had no prior experience with ceramics, shared that this led to discomfort for her: “I know nothing about ceramics…I'm literally learning alongside the kids (like), what does doing the slab mean? What does this mean?… (Y)ou know how to do some of the stuff with the machine and stuff, whereas I know nothing.” Similarly, Elizabeth, a first‐time mentor in the sewing track, expressed how her lack of sewing skills left her feeling uncertain about how to support youth working on their garments:

(W)ith sewing, I feel so far out of (it). I don't have that skill, so I can't contribute the way other mentors who have that sewing skill (can.) I think I'm struggling with that, that I just don't know how to do it, so I don't know how to help. So it's trying to just sit in with the feeling of learning with them and noticing when a youth is asking for help, but somebody hasn't picked up on that yet. Just trying to find the other little ways that I contribute has been a challenge for me.

Both mentors shared that the mismatch in skills–between their skills and the skills required to support youth–generated stress when youth reached out to them for technical support and other mentors with such expertize were unavailable. While Y‐WE encourages mentors to learn alongside youth, these situations highlighted a tension mentors felt between participating as learners and fulfilling their roles supporting youth.

Mentors with technical expertize faced a different kind of uncertainty when youth expressed project ambitions beyond the feasible scope of the program. Such uncertainty arose for these mentors when they could not reasonably bridge the instruction provided and/or competencies of the youth with the project idea or ambition of the youth participant within the duration of the program. Kat, a research team member and returning mentor with sewing experience, described the challenge of balancing support with realistic expectations: “And I think it's still hard sometimes when the youth want to start taking on their own projects like, ‘I want to make this dress,’ but we kind of just have to help them, but there isn't always a best or a right way to do it. So it feels a little bit on the fly.” As Kat noted, mentors did not want to discourage youth from pursuing their creative visions but recognized that certain projects required more guidance than the program's timeframe allowed. This mismatch between youth ambitions and the program's timeframe created a tension for mentors, who did not want to disappoint youth yet understood the limitations of what could be achieved with a weeklong program.

During the member‐checking process, four mentors from the ceramics and sewing tracks (Alexis, Shannon, Stephanie, and Rhea) expressed agreement with this interpretation. Notably, mentors in the cultural kitchen track did not report the same challenges. Helen, who mentored in this track, largely attributed this difference in experiences to how the curricula was structured across tracks and the baseline of skills mentors possessed. For example, in the cultural kitchen track, youth mostly collaborated on predetermined recipes and mentors possessed the baseline skills required to guide them, whereas in the ceramics and sewing tracks, youth's independent projects often required more individualized, skill‐specific support that not all mentors could provide.

While many mentors valued participating alongside youth, the program's model also presented specific challenges. Mentors without technical expertize to support youth often expressed stress and uncertainty. Mentors who felt like certain project ideas were impossible to complete within the duration of the program also felt pressured. Those who did not share this same stress and uncertainty did not have to support youth in individual projects and possessed the baseline skills and knowledge called upon within their tracks. While there are many important benefits to encouraging mentors to participate alongside youth participants, as demonstrated in the previous section, these challenges underscore the importance of considering the unique demands and skills of each track as well as how to effectively support mentors within a program model that utilizes a collaborative learning approach.

### 3.4 “The Many Hats of a Mentor”: The Expansive and Ambiguous Role of Mentors

All mentors were invested in cultivating a positive experience for youth participants and so wished to support other adults in the program. For some mentors, this extended to feeling a sense of responsibility to support those holding program facilitation roles, like Carmen and Kalani. In addition to providing technical assistance and social support to youth participants within the tracks and non‐track time, mentors across all tracks helped facilitators with space set‐up, project preparation, facilitation support, and clean‐up. Some mentors also provided facilitation feedback and coaching to facilitators. Although mentors for the most part felt clear on how to interact with youth participants, they expressed uncertainty about how to, or how much to, support facilitators, often fearing overstepping their roles and undermining program facilitators.

The experience of Anya, who has previously mentored in and facilitated Y‐WE programs, highlighted some of these tensions. In these previous roles, she supported the development of one of the current create facilitators while they were a youth participant of Y‐WE programs. At create this year, Anya supported this facilitator by providing guidance and feedback, being a thought partner, and stepping in to facilitate. However, Anya questioned not only the appropriateness of this mentoring work but also her approach to it:

I've mentored for a really long time, facilitated, (and) partnered with Y‐WE programs…I'm watching (Create facilitator) run a program… so it's, how do I support this person who I've been supporting in a very different way for a couple of years now in this more peer‐slash‐mentee‐mentor way of like, “Oh, you're doing a thing that at your age I started doing and I made all the same mistakes that you did and I could stop them before they happen, but would you really learn if I keep making the path so smooth for you?” And so yeah, I've just been trying to really figure out that balance.

Anya recognized where this facilitator was in their development as a facilitator given her work with them as a youth and understood the challenges they were encountering in this role. Yet, she expressed uncertainty about how to balance giving this facilitator enough space to learn by doing while also providing enough support so that they are able to succeed and youth participants have a positive experience in the program. Other mentors also noted providing targeted facilitation feedback and/or coaching to facilitators. Alexis shared providing their track facilitator feedback every time the facilitator asked, which happened to be after every session, but shared the same uncertainty about how much feedback to provide given their primary role as a mentor.

Additionally, mentors stepped in to fill perceived facilitation gaps in some cases. A returning mentor^5^ observed multiple mentors supporting a track facilitator, saying that they were “seeing all the different ways different people are supporting (the facilitator), and…kind of quietly noticing when somebody told her something and it wasn't clicking.” Another returning mentor in this same track similarly observed mentors stepping in to support the facilitator who “was not in a space…to facilitate.” This mentor noted how impactful it was to have this community of mentors present to help facilitate their track when an unexpected situation impacted the facilitator's ability to continue leading the track:

Us getting the extra hands in, us having other people to hold the container was incredibly beneficial. And I feel like, really pivoted (us) from a flaming disaster into a rollercoaster instead. Some ups and downs, but we were able to even out. So I think Y‐WE, as far as organizational things, did all of the things once things weren't going right anymore.

In a separate focus group, a mentor in a different track shared a similar observation of mentors coming together to help “hold the container” for youth participants, which highlights the expansive role of mentors within this program:

Myself and the other two mentors that were for this track, we are all teachers in schools, and so we could support (the youth) really easily and knew how to keep the energy and the momentum with their attention going and moving forward or shift it the way that we wanted to. It was very obvious that the three of us could mold the energy very easily…. Like a classroom. Versus the facilitators who have the knowledge but don't have the experience transferring the knowledge in a way that makes sense. But at the same time, the curriculum that they built was beautiful, and I'm really glad that they had folks like us, honestly, to help fill in those gaps…they needed the bridges between, and we ended up being those bridges.

In this excerpt, the returning mentor shared how the mentors in her track brought in their professional experiences as teachers into their roles of mentors. For these mentors, their prior professional experiences overall helped them be more aware of when and how to step in to support facilitators. However, mentors in this track expressed moments of uncertainty, too, about supporting facilitation because they did not want to undermine the facilitators or disrespect the intentional and “beautiful” curriculum the facilitators had designed.

Although likely not the intention of the youth‐initiated, collaborative mentoring model utilized by Y‐WE programs, it is worth noting that such a model can create ambiguity in mentors' roles. On the one hand, these expansive mentoring experiences generated stress at times, while on the other hand, encouraged mentors to critically reflect on their roles and professional work with youth in other settings. Furthermore, and perhaps most importantly, the exchange of support across the program seemed to foster a sense of community. Namely, mentors described how they provided support to other program adults but also how they felt supported by other adults in mentoring interactions and by the program more broadly. These multiple avenues of support, coupled with other intentional efforts to facilitate relational sharing, connection, and affirmation within the program, are key strengths of the program and significantly contribute to creating and sustaining a community of belonging.

## 4 Discussion

Our study found that mentoring in a weeklong summer program for racially, ethnically, and gender diverse youth enhanced the well‐being and sense of purpose of adult women mentors. This research contributes to the gap in literature about the impact of mentoring on adult mentors by qualitatively examining participants' experiences and development. The study revealed that participating in programming activities alongside youth and experiencing affirming mentor‐youth connections inspired mentors, strengthened and sustained their commitment to supporting youth, nurtured their creative agency, and enhanced their individual and collective well‐being. These experiences reflect the strengths and benefits of a collaborative and youth‐initiated mentoring model and importantly bring to light challenges mentors may encounter in such models where responsibilities extend to working with and supporting other adults in order to support youth. Findings align with existing literature on relational mentoring and radical healing.

### 4.1 Relational Mentoring, Role Uncertainty, and Well‐Being

The RCT Model of relational mentoring, which emphasizes the centrality and bidirectionality of mentoring relationships, guided this study (Fletcher et al. 2007). Three central tenets of RCT inform this framework: (1) both mentors and mentees benefit from and are influenced by mentoring relationships, (2) interactions that involve vulnerability, empathy, authenticity, and emotional competence facilitate mentors' growth in domains such as empowerment, self‐awareness, self‐worth, and relational connections in other settings, and (3) identity impacts what mentors and mentees gain from the mentoring relationship (Fletcher et al. 2007; Miller and Stiver 1997). This framework was useful for understanding the impact of mentoring relationships on women mentors within the Y‐WE program which primarily served girls, non‐binary and gender expansive youth of color.

Our results support this model, demonstrating that mentors benefit and are impacted by their experiences mentoring youth and participating in program activities alongside them. However, mentor interactions with youth did not solely lead to growth in certain domains. Rather, co‐facilitating a supportive community for youth and engaging in creative, mindful, and identity‐affirming practices alongside youth and other mentors also contributed to greater empowerment, self‐awareness, sense of purpose and connectedness among mentors. These combined experiences required not only vulnerability, empathy, authenticity and emotional competence but also on‐going self‐reflection and openness to learning, emphasizing the significance of relational connections to others but also to one's self. Furthermore, reflecting on how social conditions shaped their experiences and identification in childhood and adolescence, while experiencing a more nurturing and identity‐affirming community during the program, enhanced mentors' self‐perceptions, sense of purpose, and connections to youth.

The youth‐initiated, collaborative mentoring model embedded within the youth program offered additional unique benefits for youth and mentors. For example, youth were able to flexibly seek guidance from different mentors (and peers as well) depending on their challenges, goals, or interests, and practice engaging with various adults. For mentors, this approach distributed the responsibility of guidance and potentially reduced the overall pressure of meeting all of the needs of a youth on one's own.

Although this mentoring approach largely supported mentors' well‐being, our study found that participants' connections to others in this community may have magnified challenges they experienced. Mentors' emotional investment in the growth of youth participants, for example, may have intensified feelings of stress resulting from not having the technical skills to support them in completing their projects or from navigating uncertain expectations around supporting track facilitators. Understanding these conditions are important to helping mentorship‐based organizations recognize what expectations need to be clarified and support provided to mentors.

### 4.2 Radical Healing, Well‐Being, and Adult Women Mentors of Color

Radical healing is a framework for understanding and facilitating individual and collective well‐being of people of color and Indigenous peoples (French et al. 2020). It emphasizes a need to move beyond individual coping mechanisms to collective transformation in order to address the psychological impacts of racial trauma (Comas‐Diaz 2007; French et al. 2020; Ginwright 2018). Three core tenants of the radical healing framework–*collectivism* (collective action and community support), *radical hope* (a belief in a more just future and the possibility of significant social change), and *cultural authenticity and self‐knowledge* (connecting with cultural roots and histories)—are reflected in the experiences of mentors in this study. At Y‐WE Create, mentors contributed to creating a safe space for youth to share their experiences, assisted in the facilitation of activities that promoted a sense of belonging among youth, participated in self‐reflection exercises alongside youth, and supported youth in developing skills to create, speak about their experiences, and show up for one another.

These findings suggest that Y‐WE create and other similar mentorship‐based youth programs may serve as sites of radical healing for adult mentors working with youth. Engaging in creative practices within a supportive network empowered mentors, while participating in and helping facilitate opportunities to share personal experiences, hopes, and traditions contributed to mentors seeing their identities as sources of strength, especially when mentors interacted with youth who shared cultural backgrounds in these opportunities. Experiences like these enhanced mentors' well‐being overall and affirmed the necessity of collectivism, radical hope, strength and resistance, and cultural authenticity and self‐knowledge in both healing from and transforming unjust social conditions. Furthermore, findings highlight the significance of physical cocreation and affirming, intergenerational relationships as pathways or key sources of strength for developing a healthy sense of self and vision for the future.

### 4.3 Limitations and Implications

The valuable insights gained from this study should be considered with some limitations in mind. Although all mentors participated in focus groups, the sample size of eight and focus on a specific summer program limits generalizability of the findings. Additionally, all participants identified as women or gender minorities and people of color, which while relevant for the study, may not represent broader mentor populations. Future studies should explore the growth of mentors across different contexts and programs, specifically outside of higher education settings, to more fully capture the range of mentoring experiences.

Second, this study was conducted over the course of a week, which limits insights into the long‐term impacts of mentoring on adults. Although the participation of returning mentors and member checking process facilitated opportunities for participants to counter and expand on their experiences and growth over time, longitudinal research may enhance understanding about the long‐term impacts of mentoring on adult mentors' development.

The organization's radical inclusion values, participants' own disposition, and the understanding that the research team would anonymize data and protect participants' confidentiality likely reduced potential social desirability bias during focus group sessions. However, mentors' relationship to the organization (as current staff, former staff, and returning mentors), along with the focus group setting, may have still influenced what mentors shared with the research team. To address the influence of potential bias and assumptions, the research team systematically engaged in reflexive practice throughout the analysis of the data and facilitated a member checking process for participants to challenge findings and interpretations. Future research should further investigate the efficacy of organizational supports for mentors, such as mentor training that focuses on navigating role complexity or enhancing technical skills, in reducing stress, improving program outcomes, and predicting long‐term involvement of mentors.

Study findings offer practical considerations for organizations utilizing youth‐initiated and collaborative mentoring approaches. First, intentionally creating opportunities for mentors to participate in activities alongside youth has the potential to facilitate individual and collective well‐being of people of color. Mentorship‐based organizations working with mentors from historically marginalized communities may want to structure programmatic activities that encourage and permit mentors to deepen their understanding of their own identities, engage in creative practices, and meaningfully connect with other mentors about shared experiences. Second, mentoring experiences and relationships within the program largely enhanced mentors' well‐being, but deep emotional connection to the program community and success may intensify moments of uncertainty and stress that arise. By offering guided opportunities to clarify expectations, especially around how mentors and facilitators would like to support one another within the program, and for mentors and facilitators to reflect on and adjust planning and facilitation throughout the program, organizations can minimize the impact of challenges that may arise for mentors. Finally, developing affirming and meaningful connections with youth, within a backdrop of not necessarily having mentors that reflected their own identities and backgrounds, fostered optimism, strengthened their commitment to supporting youth and the organization, and inspired new possibilities where people of color and gender minorities are able to show up as their full selves and their creative agency is encouraged. Recognizing the need for identity‐affirming spaces for not only youth but also adults may sustain mentor involvement and growth. Considering these programmatic strategies–providing opportunities to participate in activities alongside youth, clarify expectations and improve collaboration among adults, and take part in an identity‐affirming community–can contribute to the well‐being of mentors of historically marginalized backgrounds in ways that support the development of youth.

## 5 Conclusion

This study contributes to the growing body of research on adult mentor development by demonstrating how youth mentoring can foster mentors' creative, emotional, and identity development. In co‐facilitating an identity‐affirming and transformative space for young women of color and gender diverse youth, mentors were inspired, felt more connected to a community and their sense of purpose, and engaged in creative activities that nurtured their sense of self and left them feeling more empowered. Although the program's youth‐initiated, collaborative mentoring approach largely enhanced mentors' development, uncertainty and stress did arise at times when mentors did not have the technical skills to support youth and when expectations about how to work with and support facilitators were not clarified. Despite these challenges, the mentoring approach encouraged affirming and meaningful mentor‐youth connections, which nurtured adult mentors' well‐being and sustained their commitments to supporting youth. These findings address gaps in literature, provide valuable insights for researchers in this field, and offer actionable recommendations for practitioners interested in enhancing the benefits of youth mentoring programs.

## Author Contributions

**Helen Lee:** conceptualization, methodology, data curation, investigation (equal), formal analysis (equal), software, supervision, visualization (equal), writing (lead), project administration (lead). **Janelle Salcedo:** formal analysis (equal), investigation (supporting), methodology (supporting), visualization (equal), writing (supporting), project administration (supporting). **Katharine Chen:** methodology (supporting), investigation (equal), formal analysis (equal), writing–review and editing (equal), project administration (supporting). **Amy Anderson:** formal analysis (supporting), writing (supporting), writing–review and editing (supporting).

### Peer Review

The peer review history for this article is available at https://www.webofscience.com/api/gateway/wos/peer‐review/10.1002/jcop.23182.

## Supporting information

Supporting information

## Data Availability

The data that support the findings of this study are openly available in Psych Archives at https://doi.org/10.23668/psycharchives.15170.

## Endnotes

1 Y‐WE programs center youth who identify as women, girls, trans, nonbinary, and gender expansive.

2 Y‐WE staff members who provided program support for create were also engaged in other work for their organization.

3 One mentor was only able to attend for the first 2 days of camp and so was not included in the study.

4 One mentor did not provide their age when surveyed for the study.

5 Most participant quotes in this section are not attributed to pseudonyms. This measure is intended to safeguard mentors' anonymity and to ensure that sharing their honest thoughts and experiences around mentoring challenges will not impact their relationship with Y‐WE or the Y‐WE community.
