# Supplementary material for: “This is Why We All Show Up”: How Supporting Youth Cultivates Hope, Purpose, and Well‐Being of Adult Mentors
Source: J Community Psychol. 2025 Feb 2;53(2):e23182. doi: 10.1002/jcop.23182 (PMC11788525; doi:10.1002/jcop.23182)
Supplement: Supplementary file 1 — Supporting information [file JCOP-53-0-s001.docx]

**Appendix A**

Member Checking Protocol

| Instructions for Providing Feedback on Key Findings [For Mentors] |
| --- |
| 1. Review the draft in full 2. Provide comments directly in the document after you’ve read through it once. 3. We are interested in dialoguing with you and other mentors to learn where you may agree with the data/interpretation and where you may disagree with the data/interpretation. 4. Please feel free to add questions, critiques, affirmations, corrections, and other interpretations to the document. |
| Instructions for Reviewing and Synthesizing Feedback [For Research Team Members] |
| 1. Review feedback mentors provide. 2. Summarize the feedback the mentor provided. Note any particularly interesting/useful/important insights or questions. → “Summary of Your Feedback” in follow-up meeting. 3. Note any themes/connections across feedback received and/or conversations our team has had about the study. → “Themes Across Comments” in follow-up meeting. 4. Flag any comments that are confusing/require further clarification from the mentor. Input this in the meeting agenda. → “Clarifying Questions” in follow-up meeting. 5. Prior to feedback debriefing meeting with mentor, connect with Helen/team to prepare. |
| Feedback Meeting Overview with Mentors |
| 1. (Re)introductions - 4 min    1. Research team members share names, pronouns, how they’ve been involved in the study    2. Ask mentors to share their names, pronouns, how they’ve been involved in YWE (if the research team needs this refresher)    3. Note: This is a relational and collaborative process. Feel free to build in a few minutes initially to set this stage (e.g., asking questions to get to know the participant better, check in on them, catch up, etc.). 2. Meeting Goals and Overview- 1 min    1. Dialogue with mentors about the study findings    2. Provide an opportunity for feedback and power sharing 3. Summary of Your Feedback [prepare in advance]    1. [insert summary here]    2. Does this summary reflect some of the main points you shared? 4. Themes Across Comments/Notable Connections [prepare in advance]    1. We noticed the following themes across feedback received/something you noted that our team brought up as well during the data analysis [elaborate here].    2. [prepare questions in advance] 5. Clarifying Questions [prepare in advance]    1. Finally we had a few clarifying questions regarding a few comments you left [elaborate here]. 6. Wrap-up - 5 min    1. Thank the mentor for their time!    2. Confirm compensation method/details?    3. Let them know that we will be bringing all feedback together to the team and figuring out how to incorporate the comments into our revised draft. We will reach out to them if we have follow-up questions and/or to see if they are interested in a second round of feedback. Would this be something they are interested in? |

## 
